# Targeted pH-Activated Peptide-Based Nanomaterials for Combined Photodynamic Therapy with Immunotherapy

**DOI:** 10.1021/acs.biomac.4c00141

**Published:** 2024-04-25

**Authors:** Bingbing Sun, Haowen Yang, Yudong Li, Jari F. Scheerstra, Marleen H. M. E. van Stevendaal, Shukun Li, Jan C. M. van Hest

**Affiliations:** † Bio-Organic Chemistry, Department of Chemical Engineering and Chemistry, Institute for Complex Molecular Systems, Eindhoven University of Technology Helix, P.O. Box 513, 5600 MB Eindhoven, The Netherlands; ‡ Laboratory of Immunoengineering, Department of Biomedical Engineering, Institute for Complex Molecular Systems, Eindhoven University of Technology, 5600 MB Eindhoven, The Netherlands

## Abstract

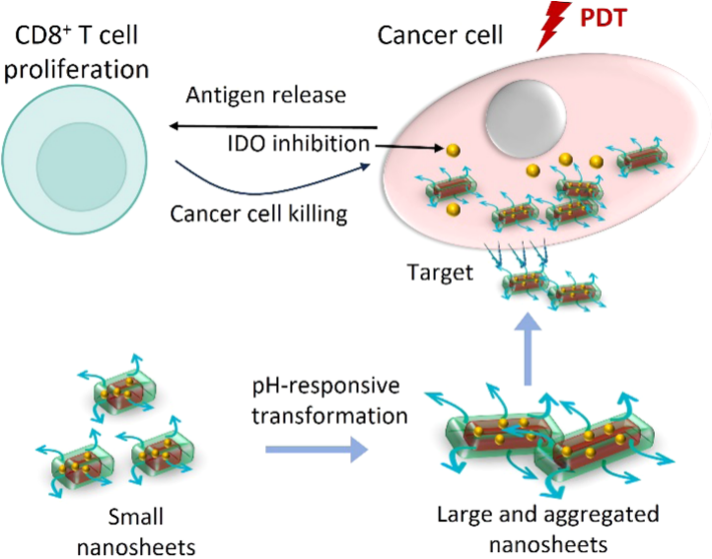

Photodynamic therapy (PDT) has demonstrated efficacy in eliminating local tumors, yet its effectiveness against metastasis is constrained. While immunotherapy has exhibited promise in a clinical context, its capacity to elicit significant systemic antitumor responses across diverse cancers is often limited by the insufficient activation of the host immune system. Consequently, the combination of PDT and immunotherapy has garnered considerable attention. In this study, we developed pH-responsive porphyrin-peptide nanosheets with tumor-targeting capabilities (PRGD) that were loaded with the IDO inhibitor NLG919 for a dual application involving PDT and immunotherapy (PRGD/NLG919). In vitro experiments revealed the heightened cellular uptake of PRGD/NLG919 nanosheets in tumor cells overexpressing αvβ3 integrins. The pH-responsive PRGD/NLG919 nanosheets demonstrated remarkable singlet oxygen generation and photocytotoxicity in HeLa cells in an acidic tumor microenvironment. When treating HeLa cells with PRGD/NLG919 nanosheets followed by laser irradiation, a more robust adaptive immune response occurred, leading to a substantial proliferation of CD3^+^CD8^+^ T cells and CD3^+^CD4^+^ T cells compared to control groups. Our pH-responsive targeted PRGD/NLG919 nanosheets therefore represent a promising nanosystem for combination therapy, offering effective PDT and an enhanced host immune response.

## Introduction

PDT, as a clinically approved phototherapy, has emerged as a viable alternative for treating cancers.^1,2^ PDT involves the utilization of a light-activated photosensitizer, oxygen, and light. While each of these components individually lacks toxic effects on biological systems, their combined action generates reactive oxygen species, resulting in localized cell death and tissue destruction.^3−6^ In contrast to conventional treatment modalities such as chemotherapy, which may lead to systemic toxicity, and radiation therapy which poses a risk to adjacent normal tissues, PDT offers several advantages.^7,8^ These include minimal invasiveness, the potential for repeated sessions without cumulative toxicity, exceptional functional and cosmetic outcomes, reduced long-term morbidity, and an improved quality of life for patients. Furthermore, PDT exhibits the capacity to induce immunogenic cell death (ICD), releasing tumor-associated antigens and damage-associated molecular patterns.^9−12^ This activation triggers the host immune system, leading to acute inflammation, leukocyte infiltration into tumors, and an enhanced presentation of tumor-derived antigens to T cells.^13^ This immunomodulatory aspect adds another layer of therapeutic benefit to PDT.

Much of PDT-related research is focused on the development of novel agents for improved tumor specificity.^14−16^ This can be achieved through two approaches. First, the modification of photosensitizers with biological conjugates, such as peptide or antibody conjugates, facilitates targeted delivery specifically to tumors.^17^ Second, the chemical conjugation of photosensitizers within delivery nanocarriers enhances the efficient transport of drugs from the administration site to the target tissue.^18,19^ Moreover, in active targeting systems, targeting ligands on the surface of nanocarriers results in increased cellular uptake by receptor-mediated endocytosis and therefore increased photosensitizer accumulation in cancer cells.^20−22^ This approach holds promise in addressing various limitations associated with photosensitizers, including a lack of tumor selectivity, poor bioavailability, and unfavorable biodistribution.^23^ Furthermore, the unique microenvironment within tumors, including elevated levels of intracellular glutathione (GSH), overexpressed enzymes, reactive oxygen species (ROS), and an acidic pH, provides an opportunity to develop specific triggers for photosensitizers to enhance tumor therapy.^24,25^ Consequently, the development of targeted stimuli-responsive photosensitizer-conjugate-based nanoparticles is highly promising for achieving targeted and effective tumor therapy.

Immunotherapy represents a promising therapeutic approach with minimal toxicity and sustained efficacy, achieved through the augmentation of the body’s immune response for precise targeting of cancer cells.^26−30^ Furthermore, it can facilitate the formation of immune memory, empowering the immune system to efficiently combat potential cancer recurrence. The successful eradication of cancer cells via the immune system hinges not only on the induction of cancer-specific T cells but also on efficacious physical interactions between T cells and cancer cells.^31^ Nevertheless, the tumor microenvironment frequently displays various inhibitory ligands and receptors on tumor cells, T cells, or antigen-presenting cells (APCs), resulting in immune suppression. Checkpoint blockade immunotherapy employs small molecules or antibodies to modulate this immunosuppressive tumor microenvironment by regulating protein expression and/or functions at dysregulated immune checkpoints.^13,32,33^ However, the immunosuppressive tumor microenvironment observed in non-T cell inflammatory tumors exhibits a limited response to immune checkpoint blockade. Indoleamine 2,3-dioxygenase (IDO), an immunoregulatory enzyme abundantly expressed in tumors, plays a pivotal role in regulating immune responses and facilitating cancer advancement. IDO catalyzes the oxidative catabolism of tryptophan (Trp) to kynurenine (Kyn). The depletion of Trp and the accumulation of the metabolite Kyn impede the differentiation and function of effector T cells while promoting the generation of regulatory T cells, exerting substantial influence on the immune response. In response to these challenges, several IDO inhibitors have been developed to curtail the activity of IDO.^34^ Despite the proven advantages of immunotherapy, a significant number of patients with various cancers show insensitivity to IDO inhibitors due to the low immunogenicity of their tumors.^35^ The antitumor immune response triggered by PDT introduces opportunities for combination therapy with immunotherapy.^36,37^ This strategy holds the potential to circumvent the escape mechanisms employed by advancing tumors to evade immune attacks, thereby enhancing therapeutic efficacy.

**Figure 1:**
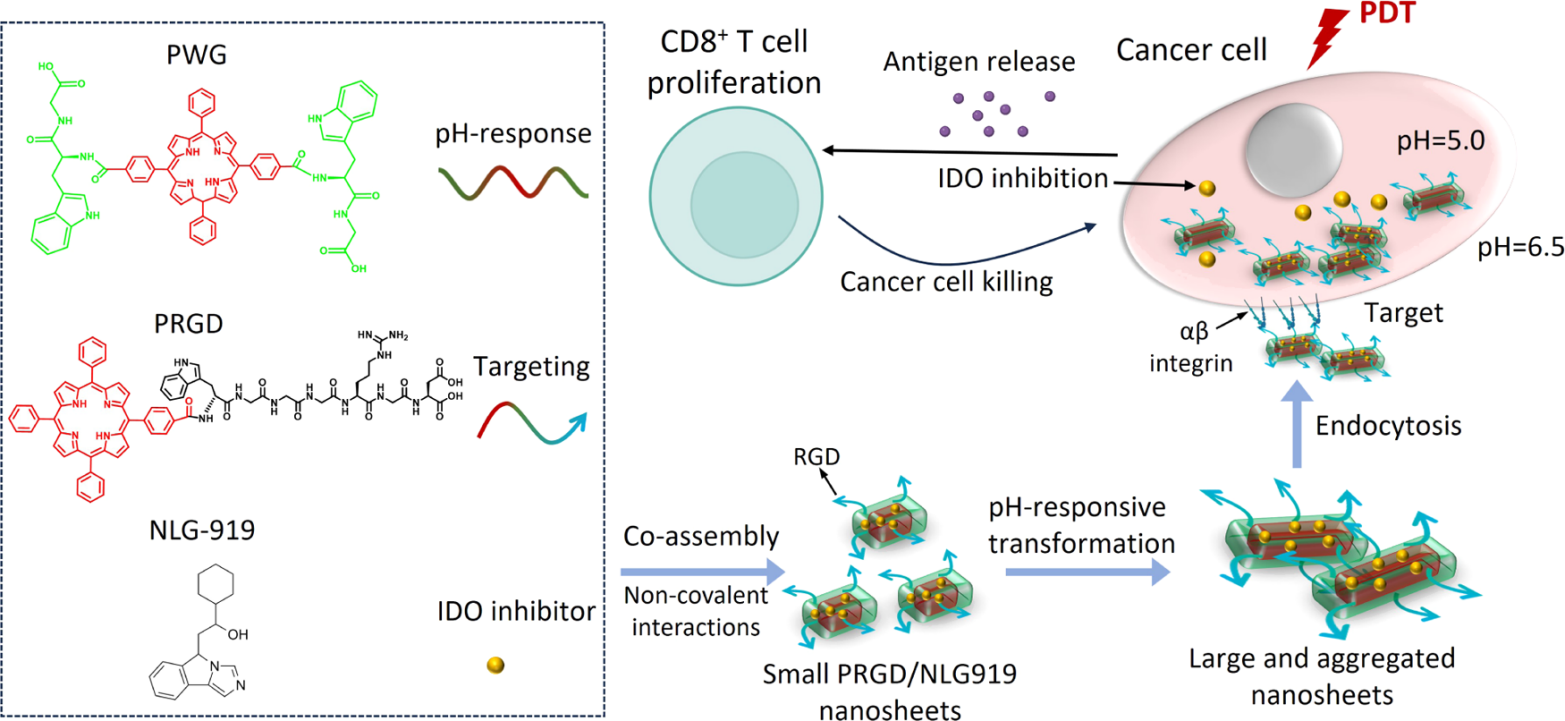
Scheme of targeted pH-activated porphyrin-peptide nanosheets for combined PDT and immunotherapy. PWG, PRGD, and NLG919 are coassembled into small PRGD/NLG919 nanosheets, which can transform into large and aggregated nanosheets at low pH (pH = 6.5 and 5.0). The RGD peptides on the surface of the nanosheets bind to the αβ integrins on the membrane of cancer cells, stimulating their uptake through endocytosis. These nanosheets can be applied for both PDT and activation of immune cells.

In this study, we designed a targeted pH-responsive nanosystem loaded with an IDO inhibitor, creating a bifunctional nanoparticle for combined immunotherapy with PDT (Figure 1). We previously developed porphyrin-peptide (PWG) nanoparticles as effective pH-responsive nanomaterials for PDT.^16^ To impart active tumor-targeting capabilities, PWG was conjugated with a targeting peptide arginine–glycine–aspartate (RGD) that specifically binds to the αβ-integrin on the tumor cell surface to form the porphyrin-peptide conjugate PWGGGRGD (PRGD). PRGD was coassembled with PWG and an IDO inhibitor (NLG919), forming a small pH-responsive nanosheet termed PRGD/NLG919. The PRGD/NLG919 nanosheets underwent a morphological transformation into aggregated sheets and displayed increased singlet oxygen generation in an acidic pH environment, mirroring the acidic conditions in the tumor microenvironment and lysosomes. This property provides them with potential for pH-triggered enhanced tumor PDT. In comparison to non-targeted PRAD/NLG control, where RGD was replaced with another peptide arginine–alanine–aspartic acid (RAD), the PRGD/NLG919 nanosheets demonstrated enhanced uptake in tumor cells, indicating their efficacy in tumor active targeting. Moreover, the PRGD/NLG919 nanosheets exhibited remarkable phototoxicity and induced enhanced proliferation of cytotoxic T lymphocytes under laser irradiation, suggesting significant efficacy in PDT and immune activation. The developed PRGD/NLG919 nanosheets therefore present a promising novel potential nanomaterial for combination immunotherapy and PDT.

## Materials and Methods

### Materials

The compound 5-(4-carboxyphenyl)-10,15,20-(triphenyl)porphyrin (98%) was procured from PorphyChem, France. Fmoc-Asp(OtBu)-Wang resin was obtained from Rapp Polymere, Germany. 9,10-Anthracenediyl-bis(methylene)dimalonic acid (ABDA), 2′,7′-dichlorofluorescein diacetate (DCFH-DA), *N*,*N*,*N*′,*N*′-tetramethyl-*O*-(1H-benzotriazol-1-yl)uronium hexafluorophosphate (HBTU), *N*,*N*-diisopropylethylamine (DIPEA), *N*,*N*′-diisopropylcarbodiimide (DIC), Fmoc-amino acids, OxymaPure (ethyl 2-cyano-2-(hydroxyimino)acetate), triisopropylsilane (TIS), trifluoroacetic acid (TFA), dimethylformamide (DMF), dimethyl sulfoxide (DMSO), diethyl ether, and all other chemicals were supplied by Sigma-Aldrich. Culture media and reagents such as Dulbecco’s modified Eagle’s medium (DMEM), fetal bovine serum (FBS), phosphate-buffered saline (PBS), Hoechst 33342, and 3-(4,5-dimethylthiazol-2-yl)-2,5-diphenyltetrazolium bromide (MTT) were procured from Thermo Fisher Scientific. Ultrapure Milli-Q water was obtained from a Labconco Water Pro PS purification system (18.2 ME).

### Porphyrin-Peptide Synthesis

Peptides were synthesized through Fmoc solid-phase peptide synthesis utilizing an automated Intavis MultiPep RSi peptide synthesizer. Fmoc-Asp(OtBu) Wang resin (0.68 mmol/g loading) served as the resin for peptide synthesis. Fmoc deprotection was achieved using 20% piperidine in DMF. Fmoc-amino acids were dissolved in DMF and sequentially coupled to the resin, employing DIPEA/HBTU (2:1) as the activator. Subsequently, 5-(4-carboxyphenyl)-10,15,20-(triphenyl)porphyrin was dissolved in DMF and coupled to the resin using DIC/OxymaPure (1:1.5) as the activator. Following synthesis, resin cleavage of the protected peptide was executed using 2.5%/2.5%/95% H_2_O/TIS/TFA, and the peptides were then precipitated in ice-cold diethyl ether. The resulting crude solids underwent five washes with diethyl ether and were subsequently lyophilized.

Purification of the peptides was carried out using a preparative reversed-phase high-performance liquid chromatography (HPLC) system, which included an LCQ Deca XP Max (Thermo Finnigan) ion-trap mass spectrometer equipped with a Surveyor autosampler and Surveyor photodiode detector array (PDA) detector (Thermo Finnigan). Solvents were delivered through a high-pressure gradient system utilizing two LC-8A pumps (Shimadzu) for the preparative system and two LC-20AD pumps (Shimadzu) for the analytical system. The crude mixture underwent purification on a reversed-phase C18 column (Atlantis T3 prep OBD, 5 μm, 150 mm × 19 mm, Waters) using a flow rate of 20 mL min^–1^ and a linear acetonitrile gradient in water with 0.1% v/v TFA. Fractions with the correct mass were collected using a PrepFC fraction collector (Gilson Inc.). The peptide solutions were collected and subjected to lyophilization.

### Characterization of Peptide-Porphyrin Conjugates

The characterization of the peptide-porphyrin conjugates involved the acquisition of ^1^H NMR spectra on a Bruker (400 MHz) spectrometer, equipped with a Bruker Sample Case autosampler. DMSOd6 served as the solvent, and TMS was employed as the internal standard. Analytical LC-MS was conducted on a C4, Jupiter SuC4300A, 150 × 2.00 mm^2^ column, utilizing a gradient of 5–100% acetonitrile in H_2_O supplemented with 0.1% v/v formic acid (FA) over a 15 min period. Matrix-assisted laser desorption/ionization time-of-flight mass spectrometry (MALDI-TOF MS) measurements were performed using an Autoflex Speed instrument (Bruker, Bremen, Germany), equipped with a 355 nm Nd:YAG smartbeam laser. This laser had a maximum repetition rate of 1000 Hz and could operate in both linear and reflector modes. The accelerating voltage was maintained at 19 kV, with a delay time set at 130 ns for all experiments. Mass spectra were acquired in the reflector positive ion mode, summing spectra from 500 random laser shots at an acquisition rate of 100 Hz. The MS spectra were calibrated using CsI clusters with known masses.

### Self-Assembly of PRGD/NLG919 Nanosheets

PWG, PRGD, and NLG919 were dissolved in DMSO at concentrations of 50, 53.6, and 15 mg mL^–1^, respectively. Subsequently, 5 μL of PWG, 4 μL of PRGD, and 5 μL of the NLG919 solution were combined in a 4 mL glass vial, followed by the dropwise slow addition of 0.5 mL of Milli-Q water under ultrasonic conditions. The preparation of PRAD/NLG919 control followed the same procedure. The nanoparticles were allowed to age for 1 day before use.

### Characterization of PRGD/NLG919 Nanosheets

Cryo-TEM experiments were conducted using a CryoTitan system (Thermo Fisher Scientific) equipped with a field emission gun and autoloader, operating at a 300 kV acceleration voltage in low-dose bright-field TEM mode. To prepare samples for cryo-TEM, grids (Lacey carbon coated, R2/2, Cu, 200 mesh, EM sciences) were glow-discharged in a Cressington 208 carbon coater for 40 s. Subsequently, 4 μL of the samples was pipetted onto the grid and blotting was performed in a Vitrobot MARK III at room temperature and 100% humidity. The grid was blotted for 3 s (offset −3) and then directly plunged and frozen in liquid ethane. Cryo-TEM images were acquired in zero-loss energy filtering mode (Gatan GIF 2002, 20 eV energy slit) using a CCD camera (Gatan model 794). Dynamic light scattering (DLS) experiments were carried out on a Malvern Z90 Zetasizer instrument equipped with a 633 nm He–Ne laser. An avalanche photodiode detector was utilized to characterize the hydrodynamic size of the particles, and scattering light at a 173° angle was detected for size and distribution analysis. The pH change of the solution was monitored using a Mettler Toledo FiveEasy Plus FEP20 pH Meter. Confocal laser scanning microscopy (CLSM) imaging of cell samples was performed with a Leica TCS SP8X.

### The pH-Responsiveness of PRGD/NLG919 Nanosheets

To explore the pH-responsiveness of PRGD/NLG919 nanosheets, a stock solution of 0.5 mg mL^–1^ nanosheets was diluted to 0.1 mg mL^–1^ using a 0.1 M phosphate buffer solution at pH 7.4, 6.5, and 5.0, respectively. The size of the nanostructures was assessed through dynamic light scattering (DLS). Fluorescence spectra (Excitation: 518 nm, Emission: 600–800 nm) and absorption spectra were recorded using a Spark 10 M microplate reader (TECAN, Switzerland). The pH-responsiveness of PRAD/NLG919 control was evaluated by using the same procedure.

### PRGD/NLG919 Nanosheets Release Profile

PRGD/NLG919 nanosheets were dispersed in PBS (1 mg of nanosheets in 2 mL of PBS). The resulting suspension was introduced into a dialysis bag with a 3500 molecular weight cutoff and dialyzed against an additional 198 mL of PBS containing 0.5% (w/v) Tween 80 (total volume 200 mL) in a beaker. Sampling was performed at 1, 2, 6, 24, and 48 h during dialysis, with 2.00 mL of the solution withdrawn from the beaker at each time point and replaced with 2 mL of fresh PBS containing 0.5% (w/v) Tween 80. The collected samples were freeze-dried to yield off-white powders, reconstituted in 0.5 mL of HPLC-grade acetonitrile/H_2_O (4:1), and subjected to filtration through a 0.2 μm PTFE filter unit for subsequent LC-MS analysis.

### Singlet Oxygen Generation

To investigate the impact of buffer pH on the photoactivity of PRGD/NLG919 nanosheets, a dispersion in water (0.5 mg mL^–1^) was diluted to 0.1 mg mL^–1^ using a 0.1 M phosphate buffer solution at pH 7.4, 6.5, and 5.0. After 12 h, the singlet oxygen probe ABDA was introduced at a final concentration of 100 μM. Subsequently, the PRGD/NLG919 nanostructure solution was exposed to a 660 nm laser (BeamQ Lasers) with a photodensity of 0.2 W cm^–2^ for 10 min. The consumption of ABDA was quantified by measuring the absorbance intensity at 378 nm over time using a Spark 10 M microplate reader (TECAN, Switzerland).

### Cell Uptake and Intracellular Trafficking of PRGD/NLG919 Nanosheets

HeLa and MCF-7 cells were seeded in an μ-Slide 8-well plate at a density of 2.5 × 10^4^ cells per well. After 24 h of culturing, the cells were treated with PRGD/NLG919 nanosheets and PRAD/NLG919 control (25 μg mL^–1^) at pH 7.4 for 0.5 h, followed by three washes with PBS. Subsequently, these cells were assessed through both CLSM and fluorescence-activated cell sorting (FACS). For experiments involving the intracellular trafficking of PRGD/NLG919 nanosheets, HeLa cells were incubated with PRGD/NLG919 nanosheets (25 μg mL^–1^) at pH 7.4 for 2 h. After incubation, the cells were stained with LumiTracker Lyso Green (150 nM) (Lumiprobe) for 5 min and Hoechst 33342 (10 μg mL^–1^) (Life Technologies) for 10 min at 37 °C. Following staining, the cells underwent three washes with PBS, were resuspended in 200 μL of Live Cell Imaging Solution, and were immediately observed using CLSM.

### Singlet Oxygen Generation In Vitro

HeLa cells were seeded in a μ-Slide 8-well plate at a density of 2.5 × 10^4^ cells per well. After 24 h of culturing, the cells were incubated with PRGD/NLG919 nanosheets at a concentration of 25 μg mL^–1^, under both pH 7.4 and 6.5 conditions for 2 h. Subsequently, the cells underwent three washes with PBS. Following this, the cells were treated with the fluorescent probe DCFH-DA (40 μM) for 30 min and Hoechst 33342 (10 μg mL^–1^) for 10 min at 37 °C in the dark. The cells were then washed three times with PBS, resuspended in 200 μL of Live Cell Imaging Solution, and exposed to a 660 nm laser for 5 min at a photodensity of 0.12 W cm^–2^. Finally, the cells were examined sequentially by using CLSM.

### Cytotoxicity Assay In Vitro

HeLa cells were plated in 96-well tissue culture plates at a density of 2.5 × 10^3^ cells per well. After 24 h of culture, the cells were exposed to varying concentrations of PRGD/NLG919 nanosheets at both pH 7.4 and pH 6.5. Following a 2 h incubation, the cells underwent three washes with PBS and were resuspended in 100 μL of DMEM. Subsequently, the cells were subjected to a 660 nm laser for 5 min at a photodensity of 0.12 W cm^–2^, followed by an additional 24 h incubation period. Cell viability was then assessed using the MTT assay with HeLa cells treated with PRGD/NLG919 nanosheets without light illumination serving as the control.

### In Vitro Immune Response of PRGD/NLG919 Nanosheets

Peripheral blood mononuclear cells (PBMCs) were isolated from buffy coats obtained from healthy donors (Sanquin, The Netherlands). HeLa cells were seeded in 6-well plates at a density of 1 × 10^5^ cells per well. Following a 24 h attachment period, fresh medium containing IFN-γ (10 ng mL^–1^) was added, and the cells were incubated for an additional 2 days. Subsequently, the medium was replaced with fresh DMEM containing 25 μg mL^–1^ PRGD/NLG919 nanosheets. After a 2 h incubation, the cells underwent three washes with PBS for 3 times and were then replaced with fresh medium. Then, laser irradiation at 660 nm (0.12 W cm^–2^, 5 min) was applied to cells. After that, PBMCs (1 × 10^6^ cells per well) were introduced along with X-VIVO medium containing l-tryptophan (0.1 mM). After 3 days of coculture, PBMCs were harvested by centrifugation at 1800 rpm for 3 min. For flow cytometry analysis, PBMC pellets were resuspended in FACS buffer and stained with CD3-FITC, CD8-PerCP, and CD4-APC/Cy7 antibodies at 4 °C for 30 min in the dark. The anti-CD3, -CD4, and -CD8-labeled PBMCs were subsequently analyzed by FACS, with data from each experiment collected at 20,000 events per sample.

### Statistical Analysis

Statistical analysis was conducted using the one-way Student’s *t* test to assess the significant impact of samples on cell viability and immune response. Significance levels were denoted as follows: *P < 0.05, **P < 0.01, ***P < 0.001, and ****P < 0.0001.

## Results and Discussion

### Synthesis and Characterization of Porphyrin-Peptides

**Figure 2:**
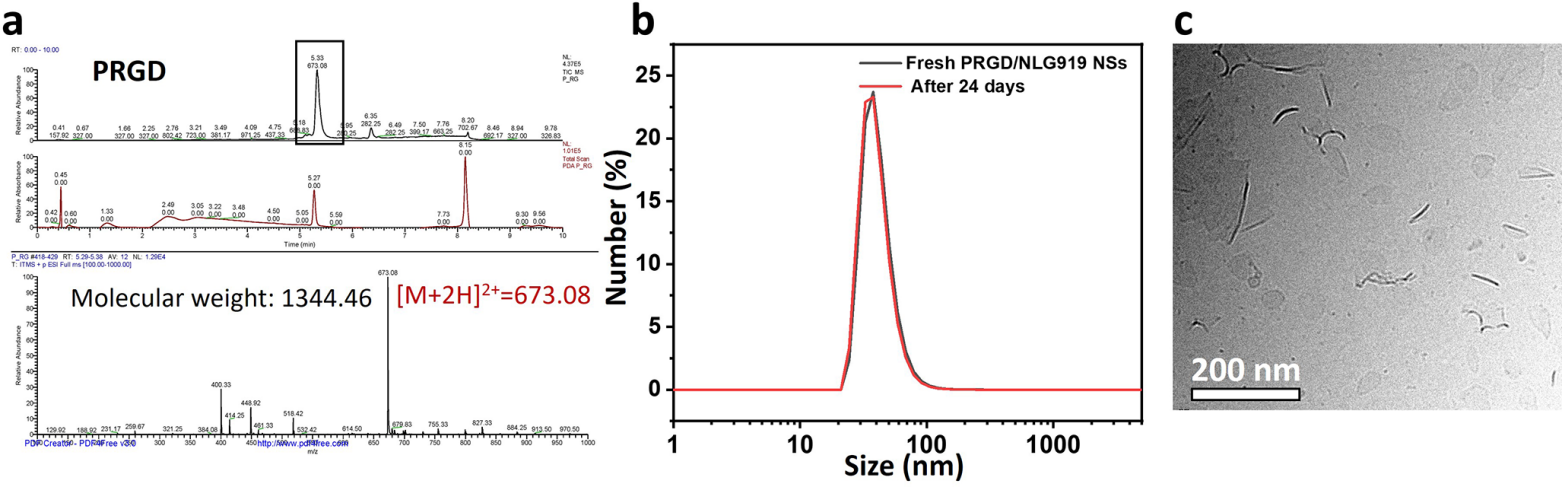
(a) LC-MS of PRGD. RT = 5.29–5.38; molecular weight: 1344.46; ESI-MS: [M + 2H]^2+^ = 673.08. (b) DLS sizes of fresh and PRGD/NLG919 nanosheets (NSs) over 24 days. (c) Cryo-TEM image of PRGD/NLG919 nanosheets in water.

RGD is a peptide ligand targeting αvβ3 integrins which are overexpressed by tumor vessels and by a wide variety of human cancer cells.^38,39^ It is therefore often used for tumor-targeting treatments. RGD was included in our recently developed peptide-porphyrin conjugate (PWG), which was shown to form pH-responsive nanoparticles capable of PDT. The targeted porphyrin-peptide PRGD consisting of a porphyrin, a linker (WGGG), as well as RGD was synthesized through solid-phase peptide synthesis. As shown in Figures 2a and S1–S2, the successful synthesis of PRGD was confirmed by LC-MS, ^1^H NMR, and MALDI-TOF MS spectroscopy after purification by HPLC. The mass detected by LC-MS and MALDI-TOF confirms the successful synthesis of PRGD, with a single peak evident in the LC-MS spectrum indicating its high purity (Figures 2a and S2). Additionally, the ^1^H NMR result further confirms the high purity of obtained PRGD (Figure S1).

### Self-Assembly and pH-Responsiveness of PRGD/NLG919 Nanosheets

**Figure 3:**
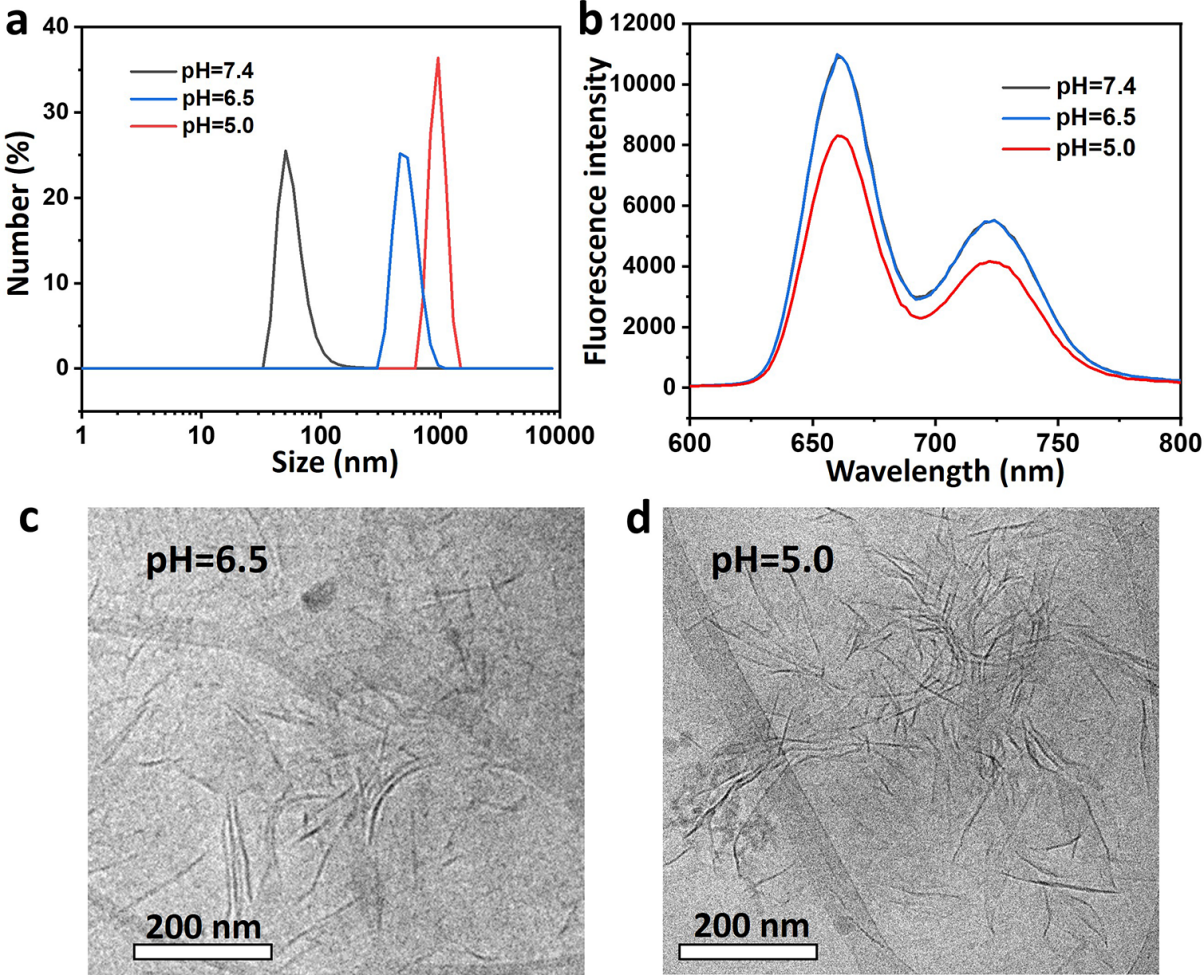
(a) DLS size and (b) fluorescence spectrum of PRGD/NLG919 nanosheets in PBS at pH = 7.4, 6.5, and 5.0. Cryo-TEM images of PRGD/NLG919 nanosheets in PBS (c) at pH = 6.5 and (d) pH = 5.0.

The noncovalent interactions between PWG, PRGD, and NLG919 for the coassembly of nanostructures involved hydrophobic interactions and hydrogen bonding, facilitated by the hydrophobic aromatic structures and functional groups (−COOH, –NH_2_, −OH) capable of forming hydrogen bonds.^16^ PWG, PRGD, and NLG919 were coassembled with a molar ratio of 5:4:6. The loading efficacy of NLG919 was 13.9 wt %. The molar ratio for PWG, PRGD, and NLG919 was selected to attain the optimal balance between stability and drug loading capacity, targeting efficacy, and immunotherapy efficacy. Figure 2b illustrates the formation of PRGD/NLG919 nanoparticles with a determined size of approximately 50 nm by using dynamic light scattering (DLS). The size of the PRGD/NLG919 nanoparticles remained unaltered for 24 days (Figure 2b), indicating their good stability for biological applications. Cryo-TEM was also applied to investigate the morphology of PRGD/NLG919 nanoparticles. Figure 2c shows that they were well dispersed into small nanosheets, each with an average length of 40 nm. Furthermore, the pH-responsive behavior of these PRGD/NLG919 nanosheets was tested by exposing them to phosphate buffer solutions at different pH. As shown in Figure 3a, the size of the nanosheets increased only slightly at pH 7.4 after 24 h incubation, confirming their stability in phosphate buffer solutions at neutral pH. However, their size increased at pH 6.5 and 5.0 and reached 356 and 952 nm after 24 h incubation, respectively. Meanwhile, the fluorescence of the nanosheets at pH = 7.4 and 6.5 was similar but decreased at pH = 5.0 (Figure 3b). This fluorescence quenching was probably caused by the pH-induced aggregation at pH 5.0.^16^ Cryo-TEM images depicted in Figure 3c,d show that these small PRGD/NLG919 nanosheets transformed into large aggregated nanosheets when exposed to acidic conditions (pH 6.5 and 5.0) for 24 h. This structural transformation might mainly be driven by enhanced intermolecular hydrogen bond formation between the protonated porphyrin-peptide molecules at lower pH, aligning with the transmorphic behavior of PWGNPs in previous work.^16^ The above results indicated that the PRGD/NLG919 nanosheets were pH-responsive and that acidic conditions (pH 6.5 and 5.0) induced their transformation into aggregated large nanosheets. The modification with RGD and the loading with NLG919 therefore did not alter the transmorphic features of the peptide-porphyrin conjugates.

### Release of NLG919 and Singlet Oxygen Generation of PRGD/NLG919 Nanosheets

**Figure 4:**
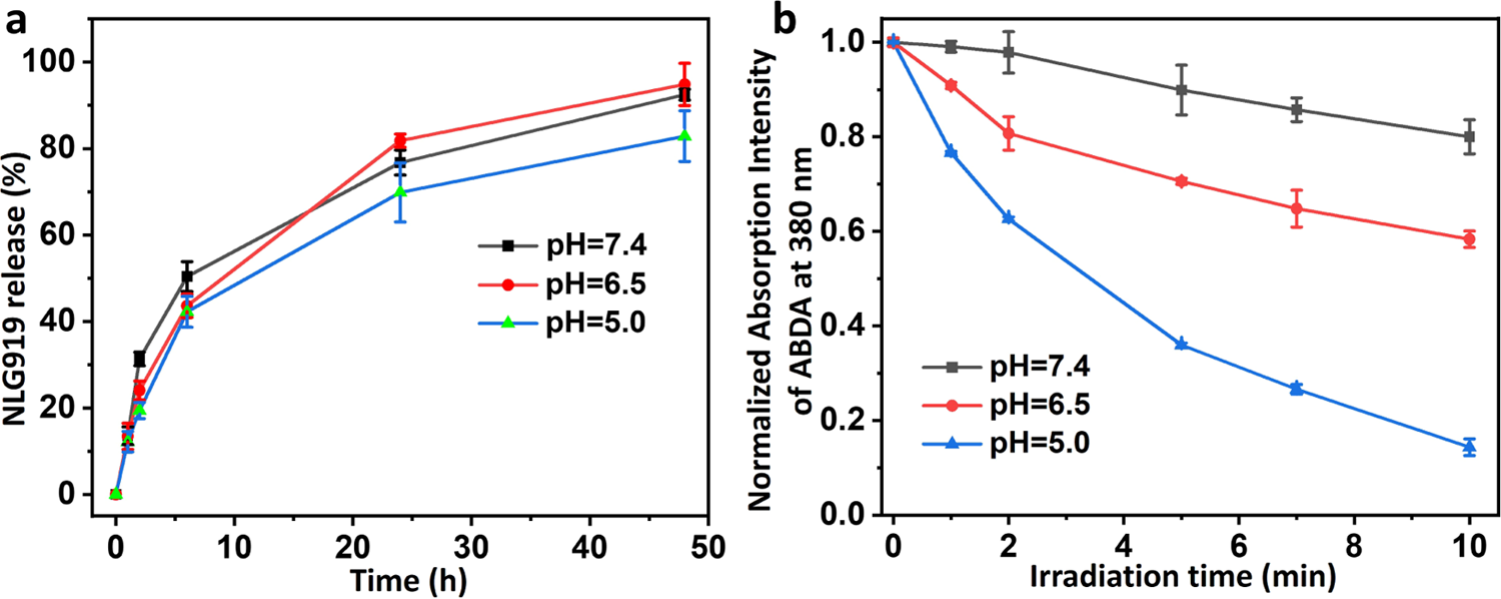
(a) Drug release of NLG919 from PRGD/NLG919 nanosheets in PBS at pH = 7.4, 6.5, and 5.0. (b) Photobleaching of ABDA by ^1^O_2_ generated by 660 nm irradiated for 10 min PRGD/NLG919 nanosheets in PBS at pH = 7.4, 6.5, and 5.0.

To understand the release dynamics of NLG919 from the pH-responsive PRGD/NLG919 nanosheets in different biological environments, this was examined in phosphate buffer solutions at pH = 7.4, 6.5, and 5.0, mimicking normal tissue, tumor microenvironment, and lysosomes, respectively. Figure 4a shows the NLG919 release profile from the PRGD/NLG919 nanosheets. The release of NLG919 at pH = 7.4, 6.5, and 5.0 was similar at 6 h, with released content of 13.6, 13.5, and 12.2%, respectively. The majority of NLG919 (76.7, 81.8, and 69.9%) was released from the nanosheets over 24 h. Notably, a slightly slower release of NLG919 at pH = 5.0 was observed, possibly attributed to the stronger aggregation of the nanosheets at this pH. Further, almost all NLG919 was released at all three pH levels after 48 h. These findings suggest that NLG919 release from PRGD/NLG919 nanosheets is effective and nearly unaffected by changes in pH, indicating their potential as reliable drug delivery systems in diverse biological environments.

The effect of pH on the photoactivity of the PRGD/NLG919 nanosheets was investigated by assessing their ability to generate singlet oxygen (^1^O_2_). This assessment employed 9,10-anthracenediylbis(methylene)dimalonic acid (ABDA) as an indicator across various pH values.^40^ As shown in Figure 4b, the bleaching of ABDA by PRGD/NLG919 nanosheets increased with the decrease of pH upon 10 min illumination with a 660 nm laser, indicating the enhanced production of ^1^O_2_ in acidic pH, which might be attributed to enhanced intersystem crossing through the structural transformation, yielding pH-activated phototoxicity of PWG.^6,16^

### Targeting Efficacy of PRGD/NLG919 Nanosheets to Tumor Cells

**Figure 5:**
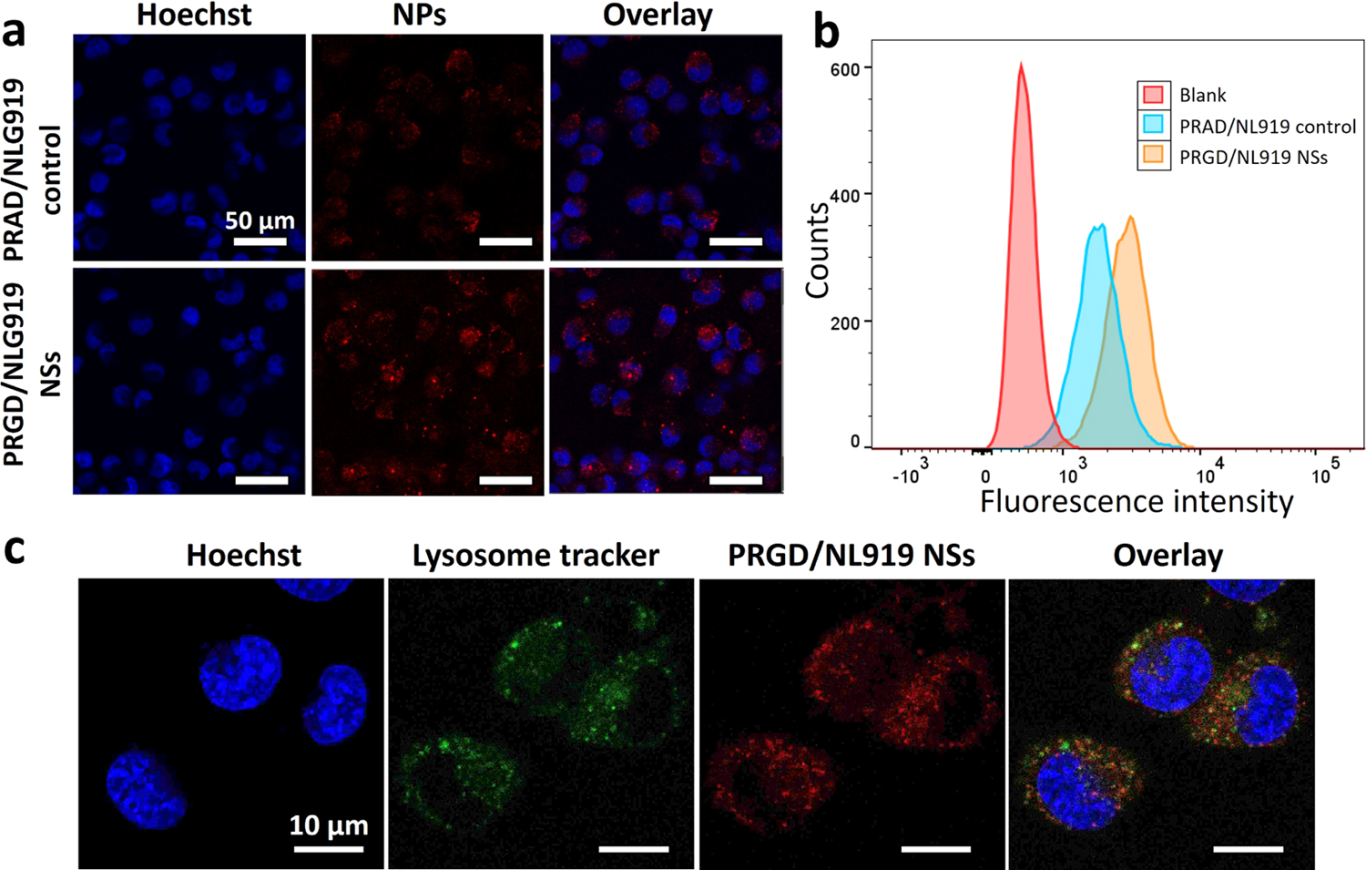
(a) Confocal images and (b) FACS result of HeLa cells treated with PRAD/NLG919 control and PRGD/NLG919 nanosheets (25 μg/mL) for 0.5 h. (c) Confocal images of HeLa cells treated with PRGD/NLG919 nanosheets (25 μg/mL) for 2 h with lysosome green tracker.

To determine the effect of targeting units displayed on the nanosheets regarding cell uptake, HeLa cells were treated with both PRGD/NLG919 nanosheets and the PRAD/NLG919 control. The PRAD/NLG919 control was prepared and exhibited physical properties similar to those of the PRGD/NLG919 nanosheets (Figures S3–S8). In Figure 5a, the CLSM images revealed a notable increase in the cellular uptake of PRGD/NLG919 nanosheets by HeLa cells compared to PRAD/NLG919. The enhanced cellular uptake of PRGD/NLG919 nanosheets in HeLa cells was further confirmed by FACS, revealing a 1.7-fold improvement compared to that of PRAD/NLG919, as shown in Figure 5b. This enhanced uptake is attributed to the specific targeting effect of the RGD ligand, which interacts with tumor cells that express elevated levels of αvβ3 integrins. Furthermore, the targeting efficacy of PRGD/NLG919 nanosheets was also investigated in αvβ3 integrin negative cells (MCF-7 cells).^41,42^ As shown in Figures S9 and S10, PRGD/NLG919 nanosheets and the PRAD/NLG919 control did not exhibit a significant difference in uptake in MCF-7 cells, which showed the effect of RGD targeting to the αvβ3 integrin receptor.

**Figure 6:**
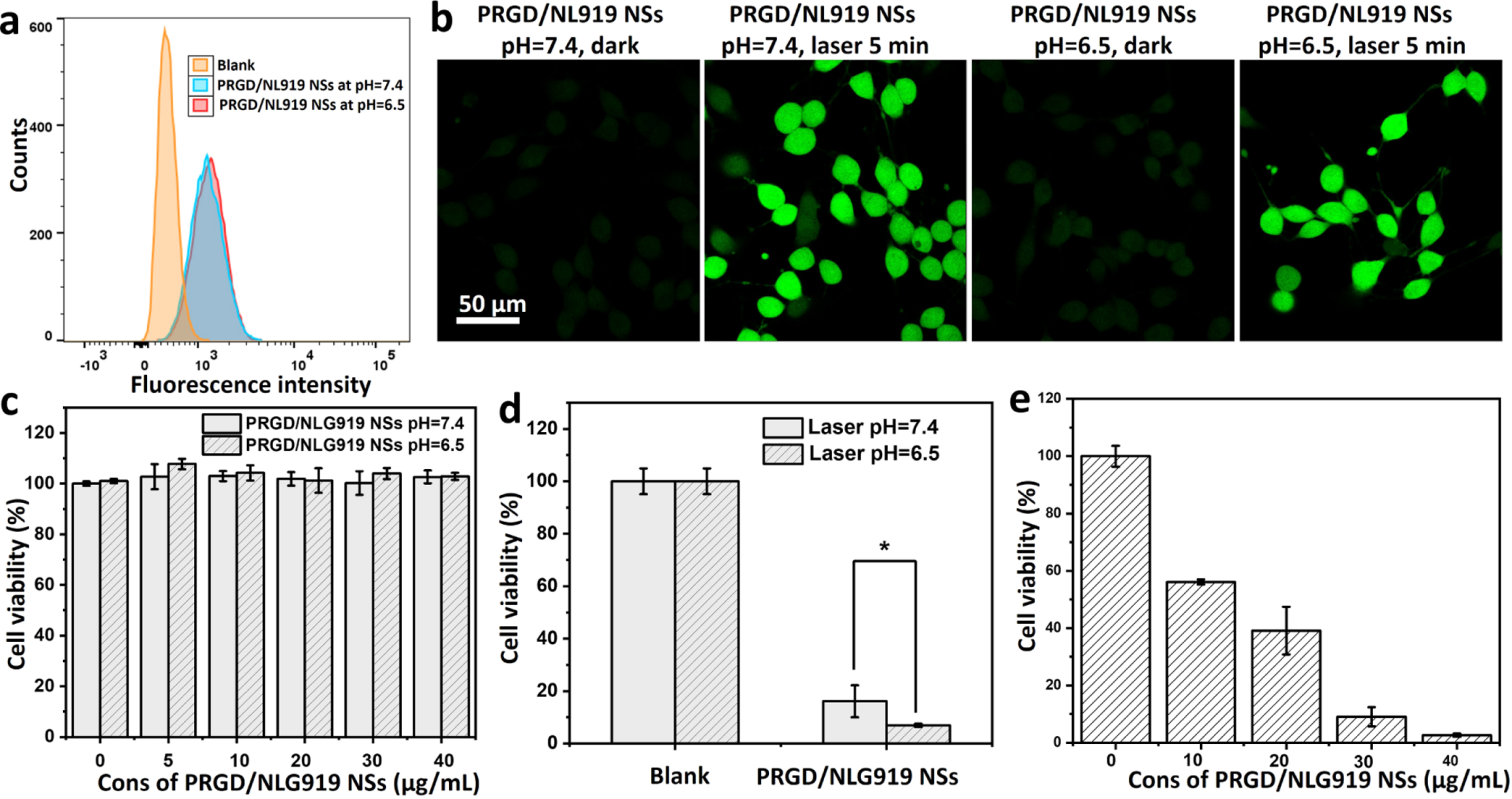
(a) FACS results of HeLa cells treated with PRGD/NLG919 nanosheets (25 μg/mL) for 0.5 h at pH = 7.4 and 6.5. (b) Intracellular singlet oxygen generation of HeLa cells cocultured with 25 μg/mL PRGD/NLG919 nanosheets for 2 h at pH = 7.4 and 6.5 with/without 660 nm laser irradiation for 5 min. (c) MTT assay of HeLa cells treated with PRGD/NLG919 nanosheets at concentrations from 0 to 40 μg/mL for 2 h. (d) MTT assay of HeLa cells cocultured with 30 μg/mL PRGD/NLG919 nanosheets for 2 h at pH = 7.4 and 6.5 under 660 nm laser irradiation for 5 min. (e) MTT assay of HeLa cells treated with NLG919 nanosheets at concentrations from 0 to 40 μg/mL for 2 h and then under 660 nm laser irradiation for 5 min at pH = 6.5.

The intracellular localization of PRGD/NLG919 nanosheets was investigated through CLSM following a 2 h incubation with HeLa cells. LumiTracker Lyso Green tracker was utilized to label lysosomes to determine the cellular distribution.^43^ In Figure 5c, a significant degree of colocalization was observed between the red fluorescence of the nanosheets and the green lysosome tracker; the Pearson correlation coefficient with lysosomes in HeLa cells was high (0.71). Given the acidic environment of lysosomes, this intracellular distribution enhances the efficacy of low pH-activated PDT. Moreover, the impact of pH on the uptake of PRGD/NLG919 nanosheets in HeLa cells was explored using FACS. As shown in Figure 6a, the fluorescence of PRGD/NLG919 nanosheets in HeLa cells was similar at both pH = 7.4 and 6.5, suggesting that the aggregation of NSs at pH 6.5 did not impede cellular uptake. Meanwhile, their aggregation in the tumor acidic microenvironment will potentially improve their accumulation and retention in tumor tissue for enhanced therapeutic efficacy in vivo, as we have shown previously.^6,16^

### In Vitro PDT

The intracellular generation of singlet oxygen by PRGD/NLG919 nanosheets was assessed using CLSM with the 2,7-dichlorofluorescein diacetate (DCFH-DA) sensor.^44^ HeLa cells were exposed to the nanosheets for 2 h and subsequently irradiated with a 660 nm laser at an intensity of 0.12 W cm^–2^ for 5 min. In Figure 6b, a marked elevation in green fluorescence was detected in HeLa cells treated with the nanosheets under laser irradiation, suggesting a heightened generation of ^1^O_2_.^45,46^ The control group exposed to identical coculture conditions but without laser irradiation did not exhibit noticeable fluorescence. This phenomenon was consistently observed at pH values of both 7.4 and 6.5 during laser irradiation, emphasizing the efficient capability of the PRGD/NLG919 nanosheets to generate ^1^O_2_ across distinct pH environments.

Then, the cytotoxicity of the PRGD/NLG919 nanosheets was assessed at pH = 7.4 and pH = 6.5 using the MTT assay.^47^ Cell viability was evaluated across varying concentrations (0, 5, 10, 20, 30, and 40 μg mL^–1^) at these two pH levels. In Figure 6c, as the concentration of the nanosheets increased, the viability of HeLa cells remained high, indicating their nontoxicity without light irradiation. When HeLa cells were exposed to a 660 nm NIR laser at an intensity of 0.12 W cm^–2^ for 5 min without nanosheet treatment, no cell death was observed either, showing the safety of the irradiation conditions for the cells. Meanwhile, HeLa cells with PRGD/NLG919 nanosheets at 30 μg mL^–1^ with 5 min laser irradiation showed a substantial decrease in cell viability to 16.1 and 7.0% at pH = 7.4 and 6.5, respectively (Figure 6d), indicating the remarkable phototoxicity. The increased phototoxicity observed at pH = 6.5, despite uptake and intracellular singlet oxygen generation comparable to that at pH = 7.4, implies a heightened sensitivity to singlet oxygen in an acidic environment. Furthermore, the phototoxicity of these PRGD/NLG919 nanosheets increased with increasing concentration from 10 to 40 μg mL^–1^ at pH = 6.5 (Figure 6e). These results show the excellent PDT of these PRGD/NLG919 nanosheets in the acidic microenvironment of tumor cells. Meanwhile, the cytotoxicity of the PRAD/NLG919 control was also assessed with and without laser irradiation. As illustrated in Figure S11, the PRAD/NLG919 control showed no toxicity to cells in the dark but exhibited phototoxicity when exposed to laser irradiation. In Figure S11, the phototoxicity of PRGD/NLG919 nanosheets was higher than that of the PRAD/NLG919 control, attributed to the enhanced cellular uptake facilitated by the targeting effect of the RGD group (Figure 5a,b).

### Immune Responses Induced by PRGD/NLG919 Nanosheets

Ensuring the production of an ample number of functional T cells, specifically CD3^+^ cells, is paramount in the context of tumor immunotherapy.^48−50^ Among these T cells, the CD3^+^CD8^+^ subpopulation, known as cytotoxic T lymphocytes (CTL), possesses the capability to effectively recognize and eliminate tumor cells, whereas the CD3^+^CD4^+^ subpopulation, termed helper T lymphocytes (Th), plays a vital role in orchestrating the antitumor immune response.^51−54^ The immune responses associated with PRGD/NLG919 nanosheets were scrutinized through FACS. In this experiment, the proliferation of both CD3^+^CD8^+^ T cells and CD3^+^CD4^+^ T cells in peripheral blood mononuclear cells (PBMCs) was assessed under various conditions: PBMC monoculture, PBMCs cocultured with PBS-treated HeLa cells, PBMCs cocultured with PRGD/NLG919 nanosheets-treated HeLa cells without or with 660 nm laser irradiation at 0.12 W cm^–2^ for 5 min (*n* = 3).

**Figure 7:**
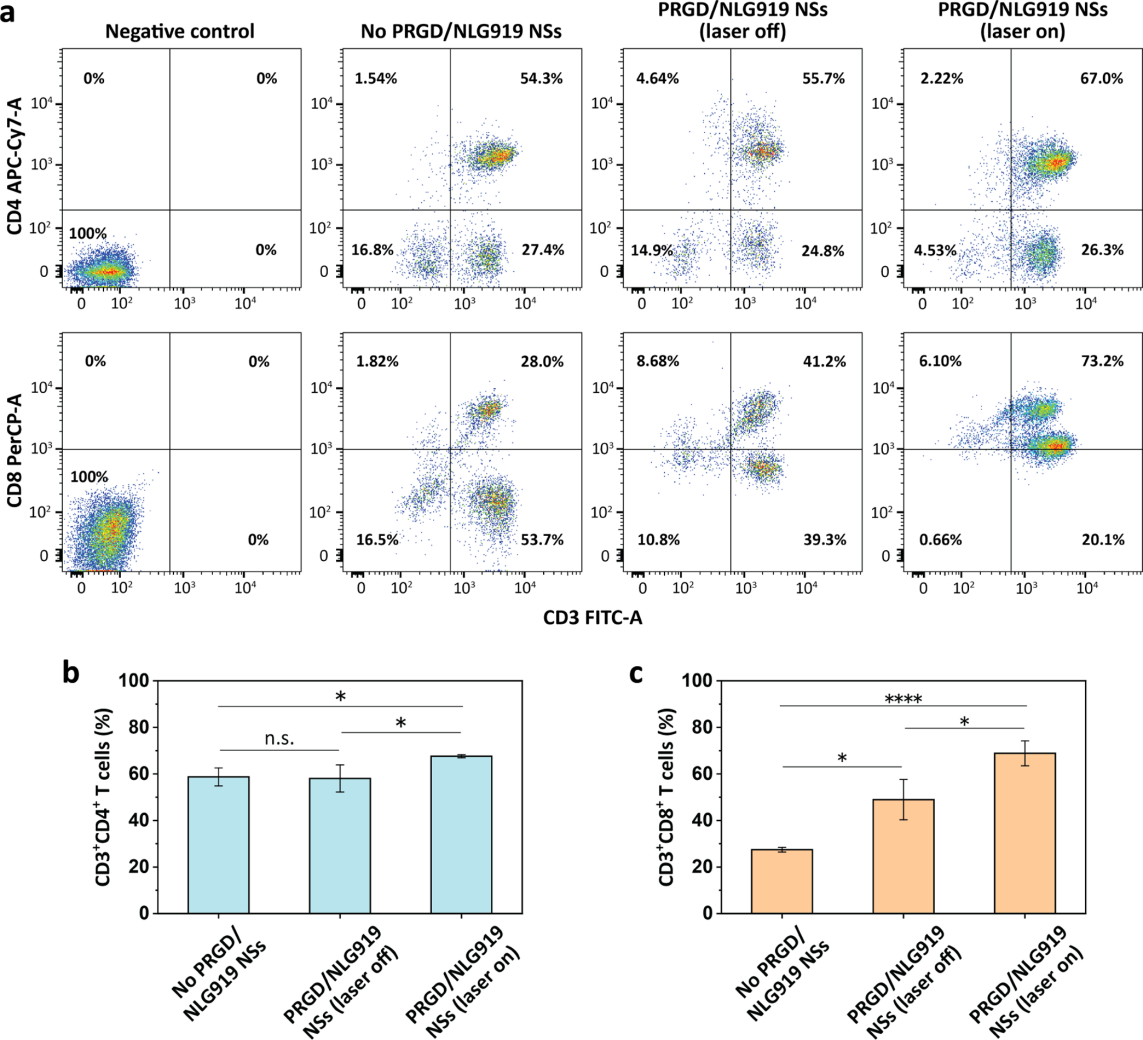
(a) FACS analysis of PBMCs under different conditions. Negative control: PBMC monoculture without antibody staining, PRGD/NLG919 NSs: PBMCs cocultured with PBS-treated HeLa cells, PBMCs cocultured with PRGD/NLG919 nanosheets-treated HeLa cells without or with 660 nm laser irradiation at 0.12 W cm^–2^ for 5 min (*n* = 3). Percentages of (b) CD3^+^CD4^+^ and (c) CD3^+^CD8^+^ T cells in PBMCs of (a). The statistically significant difference is indicated by n.s. *P* > 0.05, *P < 0.05 and ****P < 0.0001.

As shown in Figure 7a–c, the percentages of CD3^+^CD4^+^ T cells and CD3^+^CD8^+^ T cells in PBMCs cocultured with PBS-treated HeLa cells were 54.3 and 28.0%, respectively. Treatment of HeLa cells with PRGD/NLG919 nanosheets without irradiation resulted in a nonsignificant change in the percentage of CD3^+^CD4^+^ T cells (55.7%), while the percentage of CD3^+^CD8^+^ T cells increased to 41.2%, signifying an enhanced immune response induced by PRGD/NLG919 nanosheets. This results from the inhibition of IDO in tumor cells by the NLG919 released from the nanosheets, thereby activating the CD8^+^ T cells.^55^ In contrast, the percentages of both CD3^+^CD4^+^ and CD3^+^CD8^+^ T cells substantially increased (67.0 and 73.2%) upon laser irradiation (Figure 7a–c). These results indicate that the treatment of HeLa cells with PRGD/NLG919 nanosheets, especially when combined with PDT using laser irradiation, elicits stronger T-cell activation (Figure 7a–c). The substantial increase in the percentages of both CD3^+^CD4^+^ and CD3^+^CD8^+^ T cells underscores enhanced activation and proliferation of functional T-cell subsets, which are pivotal for effective antitumor immune responses.^56−58^

## Conclusions

We have designed pH-responsive targeted PRGD/NLG919 nanosheets through a coassembly process for combined PDT and immunotherapy. These nanosheets can transform into larger aggregated structures in an acidic environment, leading to an improved generation of singlet oxygen. In vitro studies demonstrated enhanced cellular uptake of PRGD/NLG919 nanosheets in HeLa cells compared with the PRAD/NLG919 control, which is attributed to the specific targeting effect of RGD on tumor cells with overexpressed αvβ3 integrins. Remarkably, the pH-responsive PRGD/NLG919 nanosheets exhibited excellent singlet oxygen generation and photocytotoxicity to HeLa cells under diverse pH conditions. Moreover, the treatment of HeLa cells with PRGD/NLG919 nanosheets under laser irradiation resulted in a significant increase in the percentage of CD3^+^CD8^+^ and CD3^+^CD4^+^ T cells in PBMCs compared to control groups, indicating a substantially enhanced adaptive immune response. Thus, our developed pH-responsive targeted PRGD/NLG919 nanosheets show great promise as a versatile nanosystem for the combination of PDT and immunotherapy due to their capability of inducing cancer cell death and eliciting a potent adaptive immune response, thereby generating synergistic anticancer effects.

## Supporting Information Available

^1^H NMR spectra, LC-MS, MALDI-TOF mass, DLS measurements, fluorescence spectrum, confocal images of PRGD/NLG919 NSs and PRAD/NLG919 control in MCF-7 cells, FACS result of PRAD/NLG919 control in MCF-7 cells, and MTT assay of HeLa cells treated with PRAD/NLG919 control (PDF)
