# Supplementary material for: Targeted pH-Activated Peptide-Based Nanomaterials for Combined Photodynamic Therapy with Immunotherapy
Source: Biomacromolecules. 2024 Apr 25;25(5):3044–54. doi: 10.1021/acs.biomac.4c00141 (PMC11094723; doi:10.1021/acs.biomac.4c00141)
Supplement: Supplementary file 1 — bm4c00141_si_001.pdf [file bm4c00141_si_001.pdf]

Targeted pH-Activated Peptide-Based Nanomaterials for Combined Photodynamic Therapy with Immunotherapy

*Bingbing Sun[+], Haowen Yang[+], Yudong Li, Jari F. Scheerstra, Marleen H. M. E. van Stevendaal, Shukun Li, Jan C. M. van Hest**

[*] Dr. B. Sun,[+] Y. Li, J. F. Scheerstra, Dr. M. H. M. E. van Stevendaal, Dr. S. Li, Prof. J. C. M. van Hest

Bio-Organic Chemistry, Department of Chemical Engineering and Chemistry, Institute for Complex Molecular Systems, Eindhoven University of Technology Helix, P. O. Box 513, 5600 MB, Eindhoven (The Netherlands)

E-mail: J.C.M.v.Hest@tue.nl

Dr. H. Yang[+]

Laboratory of Immunoengineering, Department of Biomedical Engineering, Institute for Complex Molecular Systems, Eindhoven University of Technology 5600 MB, Eindhoven (The Netherlands)

[+] These authors contributed equally to this work.

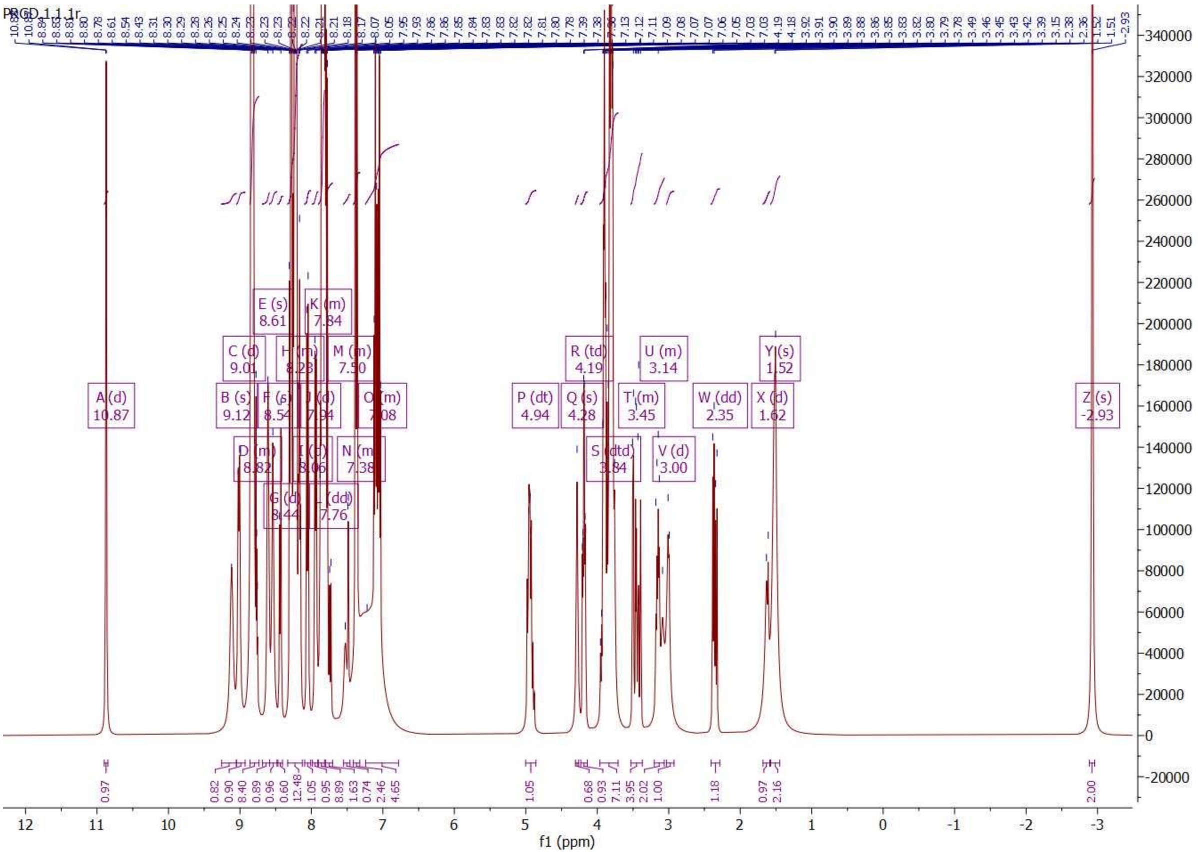

Figure S1. ^{1}H-NMR spectrum of PRGD. ^{1}H NMR (400 MHz, DMSO) δ 10.87 (d, *J* = 2.5 Hz, 1H), 9.12 (s, 1H), 9.01 (d, *J* = 7.9 Hz, 1H), 8.86 – 8.73 (m, 8H), 8.61 (s, 1H), 8.54 (s, 1H), 8.44 (d, *J* = 7.7 Hz, 1H), 8.33 – 8.13 (m, 12H), 8.06 (d, *J* = 7.6 Hz, 1H), 7.94 (d, *J* = 7.4 Hz, 1H), 7.90 – 7.82 (m, 9H), 7.76 (dd, *J* = 20.2, 7.8 Hz, 2H), 7.55 – 7.46 (m, 1H), 7.41 – 7.32 (m, 2H), 7.24 – 6.78 (m, 5H), 4.94 (dt, *J* = 14.0, 9.2 Hz, 1H), 4.28 (s, 1H), 4.19 (td, *J* = 7.6, 3.3 Hz, 1H), 3.84 (dtd, *J* = 41.2, 10.1, 6.9 Hz, 7H), 3.53 – 3.37 (m, 4H), 3.20 – 3.06 (m, 2H), 3.00 (d, *J* = 6.6 Hz, 1H), 2.35 (dd, *J* = 16.0, 7.9 Hz, 1H), 1.62 (d, *J* = 9.8 Hz, 1H), 1.52 (s, 2H), -2.93 (s, 2H).

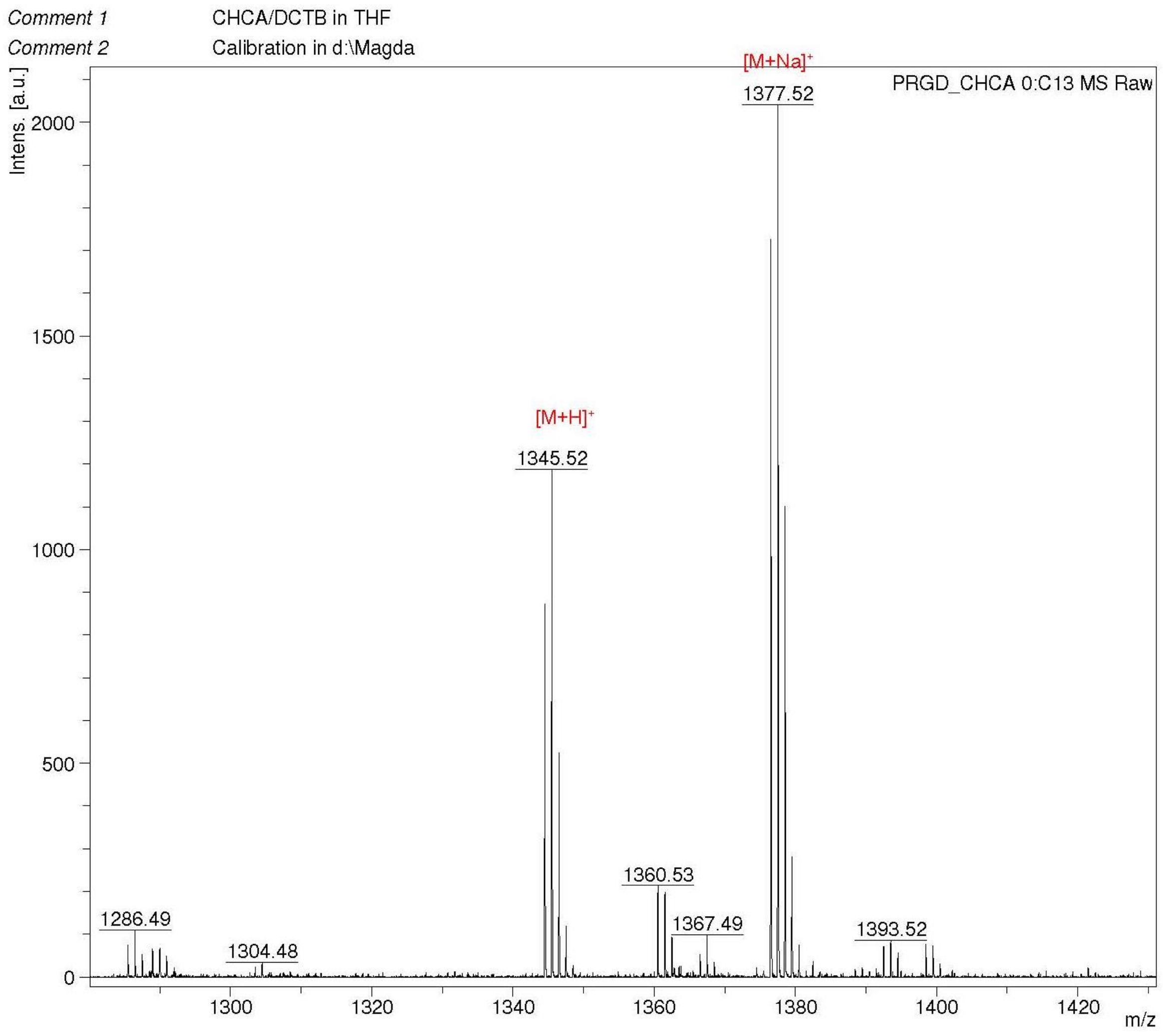

Figure S2. MALDI-TOF mass analysis of PRGD. m/z, $[M+H]^+$, 1345.52.

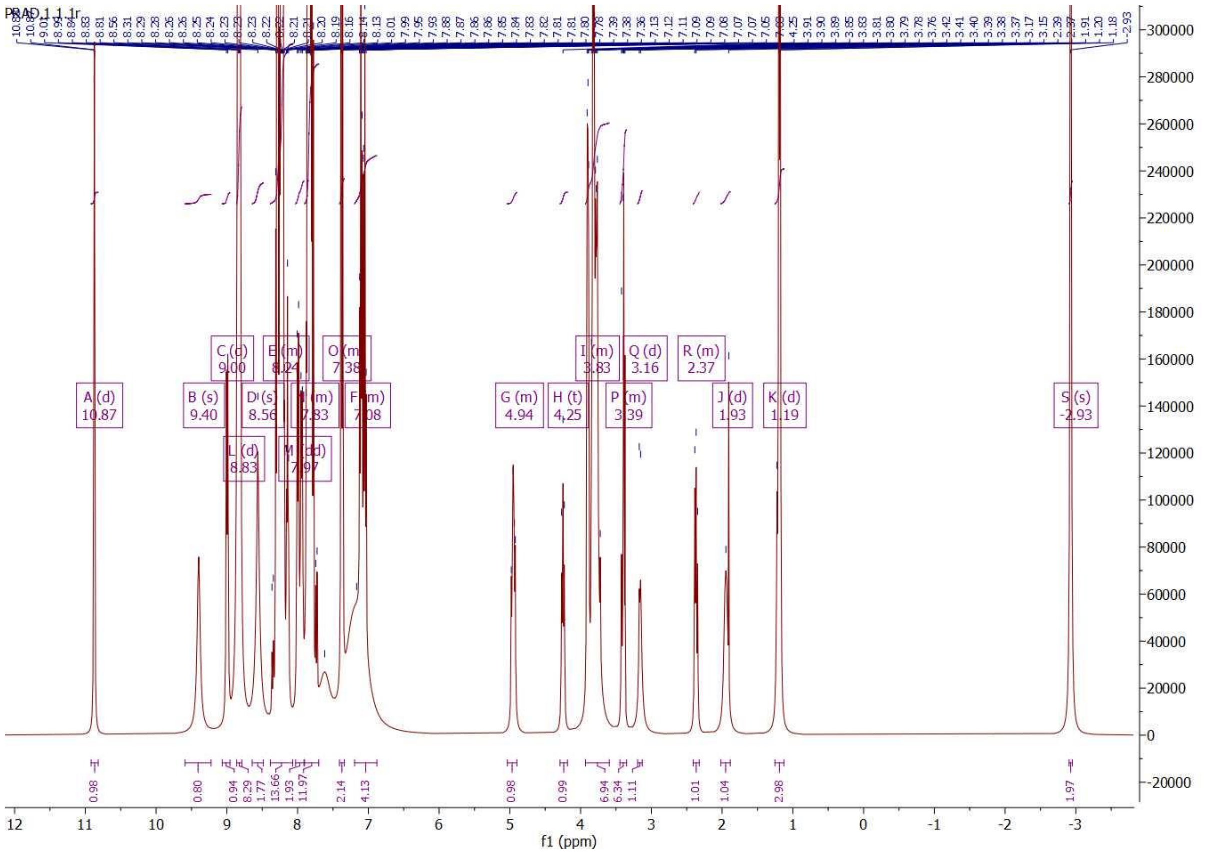

Figure S3. ^{1}H-NMR spectrum of PRAD. ^{1}H NMR (400 MHz, DMSO) δ 10.87 (d, *J* = 2.5 Hz, 1H), 9.40 (s, 1H), 9.00 (d, *J* = 7.7 Hz, 1H), 8.83 (d, *J* = 4.8 Hz, 8H), 8.56 (s, 2H), 8.38 – 8.07 (m, 14H), 7.97 (dd, *J* = 21.3, 7.6 Hz, 2H), 7.90 – 7.70 (m, 12H), 7.41 – 7.34 (m, 2H), 7.19 – 6.88 (m, 4H), 5.04 – 4.90 (m, 1H), 4.25 (t, *J* = 7.6 Hz, 1H), 3.93 – 3.59 (m, 7H), 3.44 – 3.35 (m, 6H), 3.16 (d, *J* = 7.7 Hz, 1H), 2.41 – 2.32 (m, 1H), 1.93 (d, *J* = 16.1 Hz, 1H), 1.19 (d, *J* = 7.2 Hz, 3H), -2.93 (s, 2H).

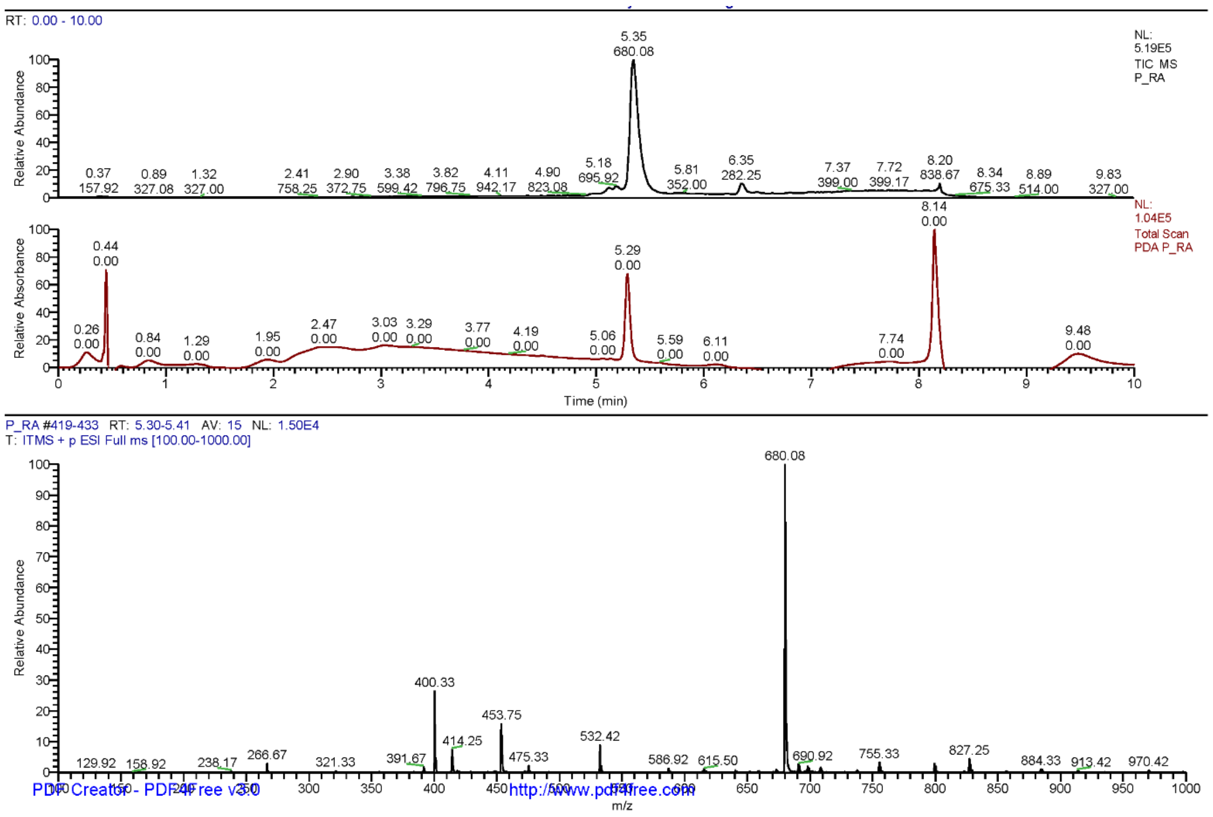

Figure S4. LC-MS of PRAD, RT=5.30-5.41; Molecular weight: 1358.49; ESI-MS: $[M+2H]^{2+}$ =680.08.

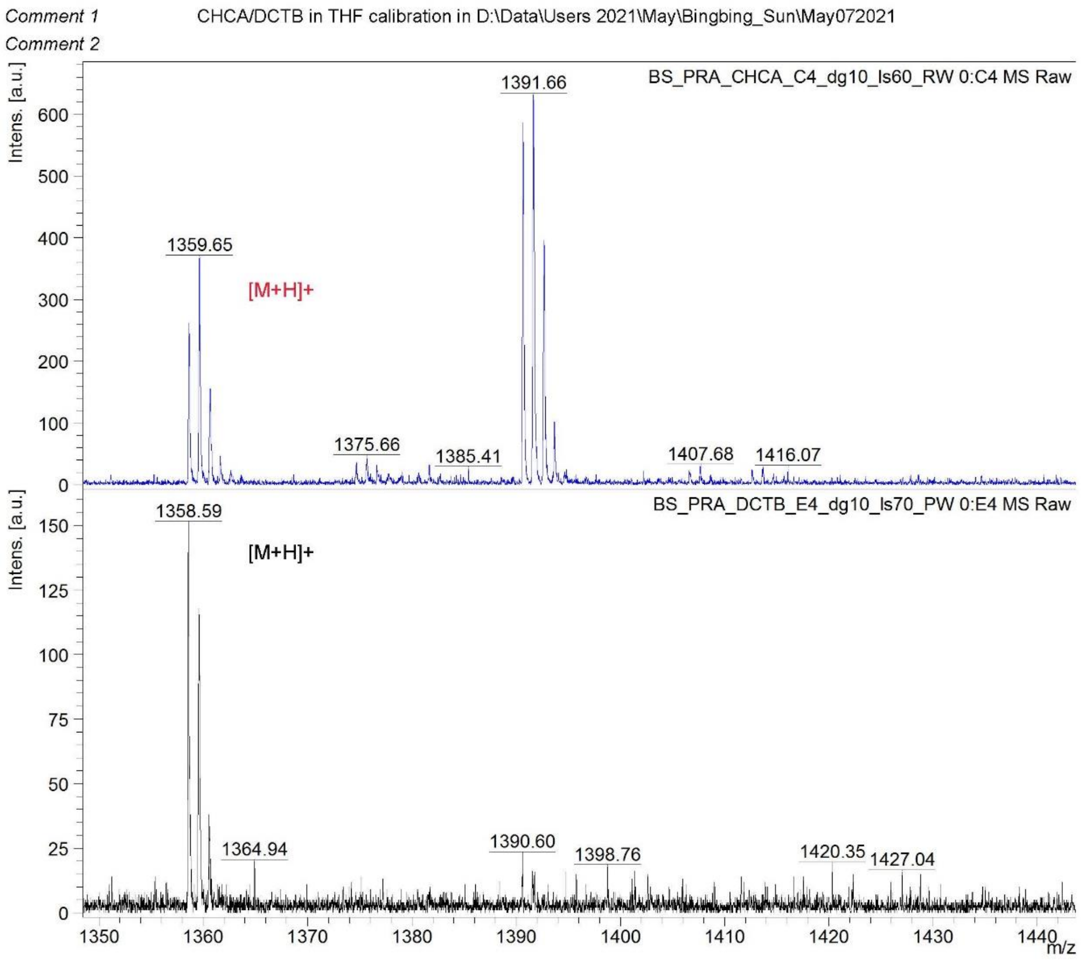

Figure S5. MALDI-TOF Mass analysis of PRAD. m/z, $[M+H]^+$, 1359.65.

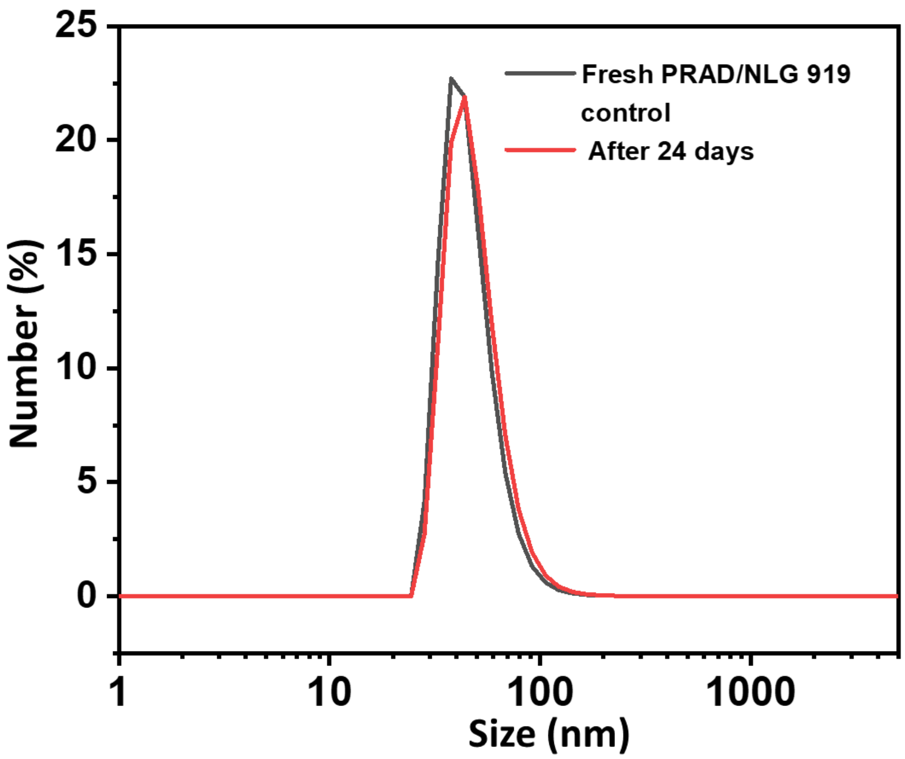

Figure S6. DLS sizes of fresh and 24 days aged PRAD/NLG919 control.

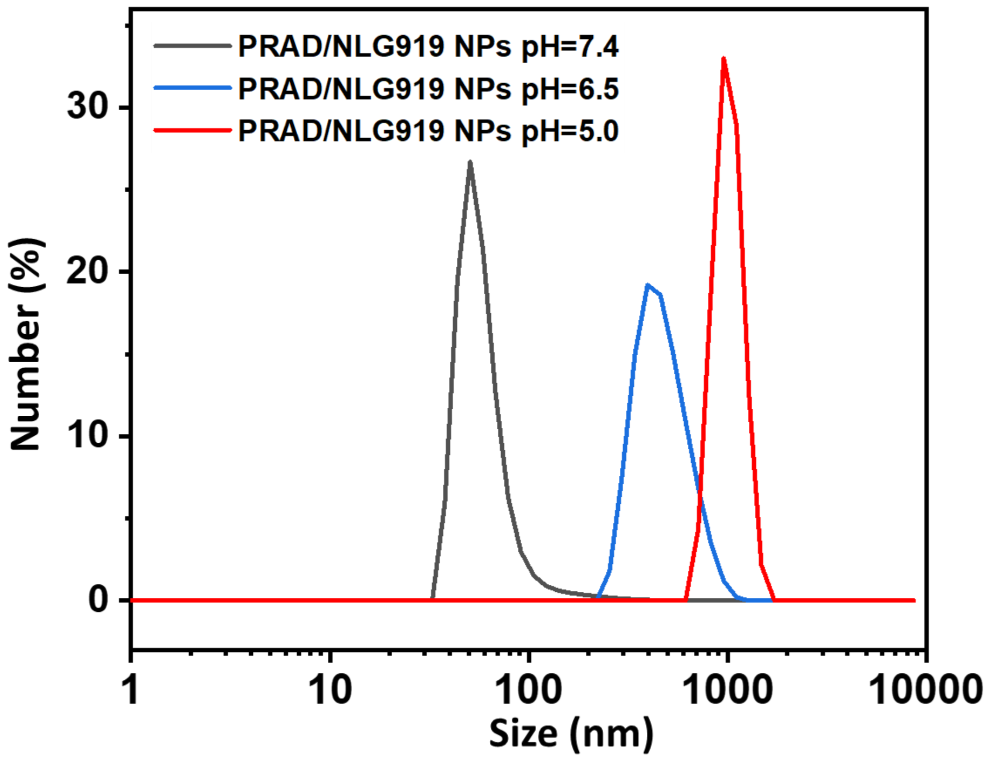

Figure S7. DLS size of PRAD/NLG919 control in PBS at pH=7.4, 6.5 and 5.0.

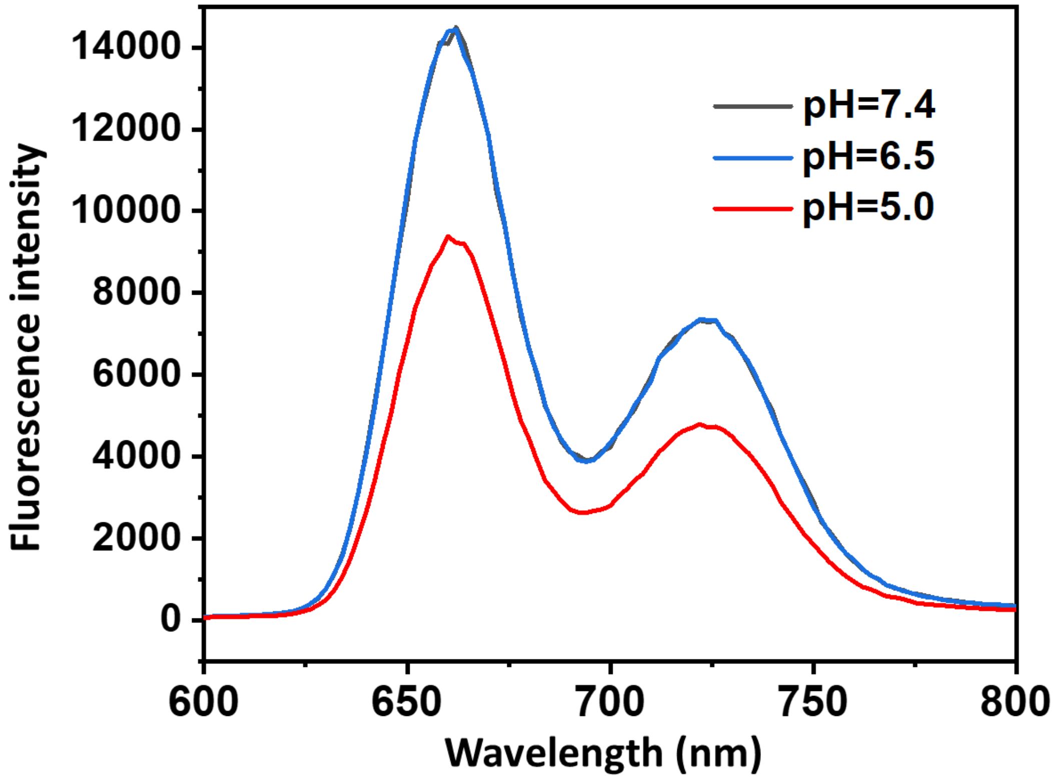

Figure S8. Fluorescence spectrum of PRAD/NLG919 control in PBS at pH=7.4, 6.5 and 5.0.

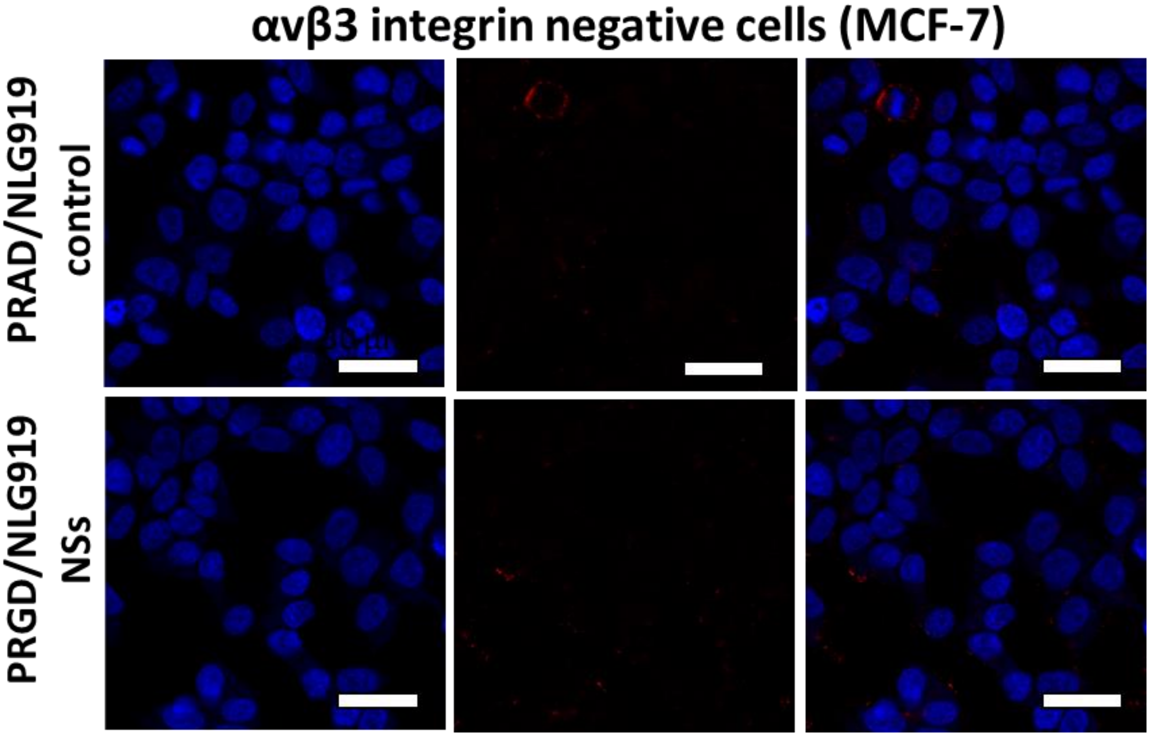

Figure S9. Confocal images of MCF-7 cells were treated with PRAD/NLG919 control and PRGD/NLG919 nanosheets (25 μg/mL) for 0.5 h.

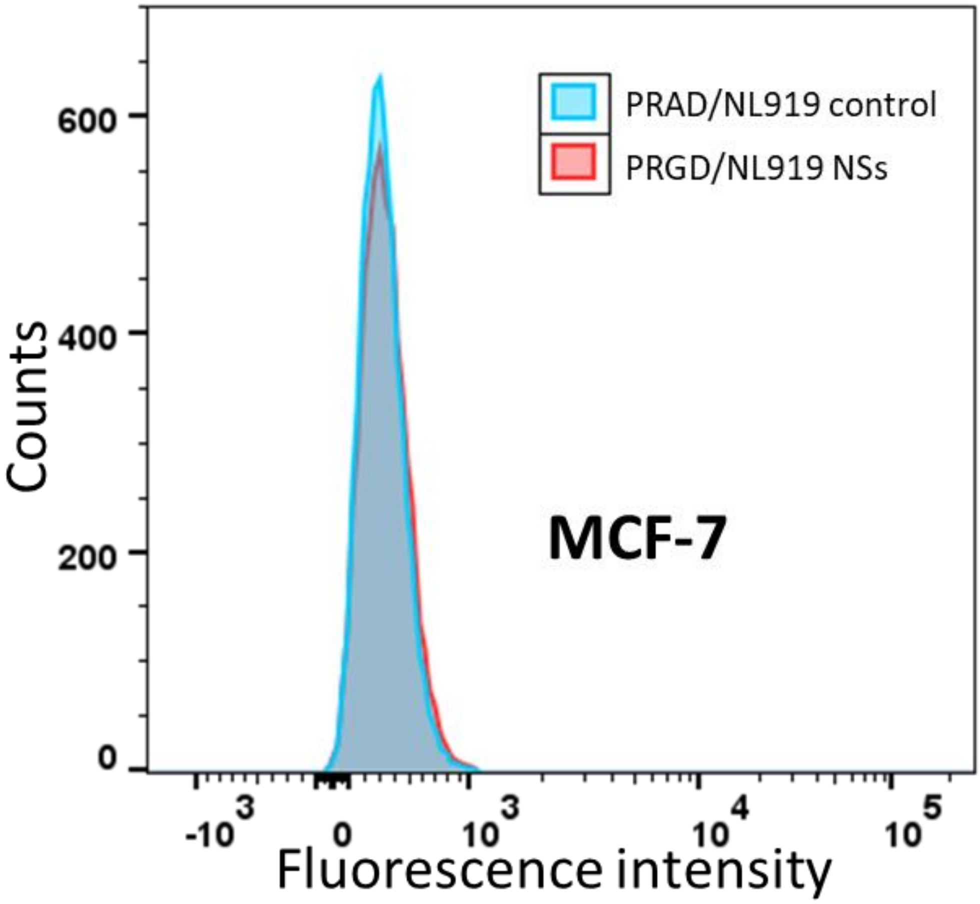

Figure S10. FACS result of HeLa cells treated with PRAD/NLG919 control and PRGD/NLG919 nanosheets (25 µg/mL) for 0.5 h.

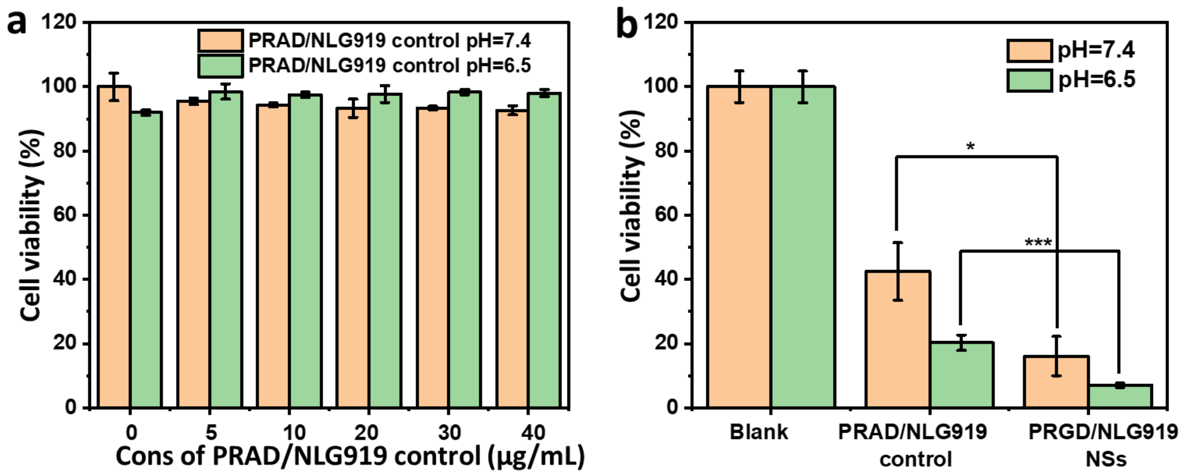

Figure S11. (a) MTT assay of HeLa cells treated with PRAD/NLG919 control at concentrations from 0 to 40 μg/mL for 2 h. (b) MTT assay of HeLa cells cocultured with 30 μg/mL PRGD/NLG919 nanosheets and PRAD/NLG919 control for 2 h at pH=7.4 and 6.5 under 660 nm laser irradiation for 5 min.
